# GENERATIVITY ACROSS ADULTHOOD: THE EFFECTS OF AGE AND AFFECT

**DOI:** 10.1093/geroni/igz038.2842

**Published:** 2019-11-08

**Authors:** Niccole A Nelson, Cindy Bergeman

**Affiliations:** University of Notre Dame, Notre Dame, Indiana, United States

## Abstract

Generativity, or investment in the next generation, is heavily implicated in successful aging. Due to its benefits to both the individual and society, researchers have attempted to identify factors that contribute to generativity. Most studies on generativity, however, have been conducted in cross section, limiting established relations to those occurring between individuals, not within them. The identification of true within-person effects is vital, as between-person relations do not accurately describe within-person processes. One factor that may contribute to generativity within individuals is positive affect (PA), as experiencing PA seems to broaden individuals’ resources, and ultimately build human flourishing. This process may also be moderated by age or change over time. To test these ideas, the current study applied multilevel modeling to ten years of data from N = 1,117 mid-to-later life individuals. A main effects model indicated between-person effects such that younger individuals, as well as individuals with higher global PA, tended to report higher generativity, on average, than older individuals and individuals with lower global PA, respectively. Next, considering within-person effects, individuals tended to report higher generativity during years they experienced more PA. Finally, a two-way interaction effect indicated an age-related difference in a within-person process: although midlife individuals reported higher generativity during years they experienced more PA, later-life individuals did not show this effect. Theoretical implications, including age-graded goal strivings and the broaden-and-build theory, will be discussed.

